# Narcissism in independent and interdependent cultures

**DOI:** 10.1016/j.paid.2021.110716

**Published:** 2021-03-12

**Authors:** Emanuel Jauk, Dorothee Breyer, Philipp Kanske, Akio Wakabayashi

**Affiliations:** a Clinical Psychology and Behavioral Neuroscience, Faculty of Psychology, Technische Universität Dresden, Dresden, Germany; b Department of Psychology, University of Graz, Graz, Austria; c Max Planck Institute for Human Cognitive and Brain Sciences, Leipzig, Germany; d Department of Psychology, Chiba University, Chiba, Japan

**Keywords:** Grandiose narcissism, Vulnerable narcissism, Independent self-construal, Interdependent self-construal, Cross-cultural research

## Abstract

Narcissism can manifest in a grandiose form – admiration-seeking, exhibitionism, and dominance – or a vulnerable form – anxiety, withdrawal, and hypersensitivity. While grandiose narcissism is conceptually in line with an independent self-construal, as prevalent in Western countries, the vulnerable form can be assumed to relate more to an interdependent self-construal, as prevalent in Eastern countries. We studied both forms of narcissism in Germany and Japan (*N*s = 258, 280), which differ fundamentally in their independent and interdependent self-construal, yet are similar regarding global developmental standards. We tested whether (1) mean differences in both narcissism forms would conform to the predominant self-construal, (2) self-construal would explain variance in narcissism beyond broad personality traits, and (3) there would be stronger mental health tradeoffs for culturally incongruent forms of narcissism. Our results largely confirm these expectations for vulnerable narcissism, which is (1) more prevalent in Japan than Germany, (2) related to self-construal beyond broad traits, and, (3) more strongly related to mental health problems in Germany than Japan. For grandiose narcissism, data analyses indicated that construct equivalence can only be assumed for the entitlement factor, and internal structure and nomological networks differ substantially between cultural contexts.

## 1 Introduction

### 1.1 Grandiose and vulnerable narcissism

Narcissism is a personality trait with two faces – grandiosity and vulnerability (Wink, 1991). While both are characterized by feelings of self-importance and entitlement (Krizan & Herlache, 2018; Weiss et al., 2019), grandiosity and vulnerability constitute different phenotypes: grandiose narcissism is characterized by admiration-seeking, exhibitionism, and dominance, whereas vulnerable narcissism is characterized by anxiety, withdrawal, and hypersensitivity (Krizan & Herlache, 2018; Miller et al., 2016; Weiss et al., 2019). In terms of the Five-Factor-Model (FFM), grandiose and vulnerable narcissism can be described as agentic-antagonistic and neurotic-antagonistic personality styles (Miller et al., 2016; Weiss et al., 2019). Grandiose and vulnerable narcissism build on different nomological networks and are weakly related or unrelated in the general population, supporting the idea of distinct phenotypes (Jauk et al., 2017; Jauk & Kaufman, 2018). Beyond that, grandiose narcissism is largely associated with self-reports of good mental health, whereas vulnerable narcissism is associated with psychological maladjustment (Kaufman et al., 2018).

### 1.2 Narcissism as a Western concept

The concept of narcissism is clearly rooted in Western cultures: originating in Greek mythology 2000 years ago, it was picked up by psychodynamic theorists in central Europe at the beginning of the twentieth century (e.g., Freud, 1914), and listed as a mental disorder in the United States in 1980 (American Psychiatric Association, 1980). In the past decades, a lively debate flourished on the question whether and how (grandiose) narcissism is tied to cultural changes in Western countries (e.g., Donnellan et al., 2009) or different sociopolitical systems within the Western world (Vater et al., 2018). While this research highlights that the individual is situated in a cultural context, and changes in this context also echo in individual personality (Vater et al., 2018), there is to date little research investigating narcissism across fundamentally different cultures, thereby also stepping beyond the cultural context in which the concept originated. Moreover, to our knowledge, existing cross-cultural research on narcissism has exclusively focused on its grandiose form, disregarding variation in its vulnerable form. This distinction might be vital for cross-cultural comparisons, as we discuss in the following.

### 1.3 Independent and interdependent self-construal

A self-construal is a “constellation of thoughts, feelings, and actions concerning one’s relationship to others, and the self as distinct from others” (Singelis, 1994, p. 581). The independent self-construal, as prevalent in most Western cultures, emphasizes (1) internal features such as individuals’ abilities, thoughts, and feelings, (2) uniqueness of the individual, (3) individual goal-orientation, and (4) direct communication (i.e., expressing own thoughts or needs in an outright manner). The interdependent self-construal, as more prevalent in Eastern cultures, in contrast values (1) external features such as status, roles, and relationships, (2) belonging and fitting in, (3) knowing one’s proper place and action, and (4) indirect communication (i.e., attuning to the anticipated mental states of others; Markus & Kitayama, 1991; Singelis, 1994). Independent self-construal and interdependent self-construal are conceptualized as separate dimensions rather than opposites, meaning that individuals can vary on both dimensions. Individuals in Western cultures typically display high independent and low interdependent orientation, whereas the opposite is true for individuals in Eastern cultures (Singelis, 1994). However, irrespective of these general trends, there are also major cultural differences within Western and Eastern cultures, why the country-level might be a more appropriate level of analysis than comparing “Western” and “Eastern” cultures (Matsumoto, 1999). We investigate grandiose and vulnerable narcissism across Germany and Japan. Germany, as a Western-European country, displays a predominantly independent self-construal (similar to North America; Markus & Kitayama, 1991), whereas Japan is the most prototypical example of an interdependent self-construal (Markus & Kitayama, 1991, 2010).^1^ Beyond that, both countries are similar in global developmental standards (Conceição & United Nations Development Programme, 2019), making them well suited for cross-cultural comparisons.

### 1.4 Narcissism and self-construal

Research based on Western samples found that grandiose narcissism is positively associated with independent self-construal, and either negatively correlated or uncorrelated with interdependent self-construal (Konrath et al., 2009; Rohmann et al., 2012). Vulnerable narcissism, in contrast, is not associated with independent, but positively correlated with interdependent self-construal (Rohmann et al., 2012). While the agentic-antagonistic interpersonal style associated with grandiose narcissism seems well in line with an independent self-construal (emphasizing characteristics of the individual, their perceived uniqueness, and individual goals), the association between vulnerable narcissism and interdependent self-construal may require a closer look: vulnerable narcissism is characterized by marked social insecurity (e.g. Pincus et al., 2009; Wink, 1991), particularly facing potential interpersonal rejection (Besser & Priel, 2010), and a heightened proneness to experiencing shame (Pincus et al., 2009; Poless et al., 2018). An interdependent self-construal that emphasizes belonging and fitting in, with shame being a distinctive characteristic (Benedict, 1989), might thus give rise to the vulnerable rather than the grandiose form of narcissism. Based on the country-level differences in self-construal discussed above, we hypothesize that grandiose narcissism will be higher in Germany, whereas vulnerable narcissism will be higher in Japan, and that these differences can be attributed to differences in self-construal.

### 1.5 Narcissism, self-construal, and mental health

Within Western cultures, grandiose narcissism is largely (though not exclusively) associated with self-reports of good mental health, whereas vulnerable narcissism is associated with pronounced psychological maladjustment (Kaufman et al., 2018). Independent and interdependent self-construal are also differentially associated with mental health across Western and Eastern cultures in the way that *personal control* is related to good mental health in Western cultures whereas *relational quality* is related to good mental health in Eastern cultures (Kitayama et al., 2010). Thus, it could be hypothesized that grandiose narcissism, indicative of high feelings of personal control (agency; Weiss et al., 2019), will show positive associations with mental health-related outcomes that are relatively stronger in Germany than in Japan. Vulnerable narcissism, indicative of low feelings of social connectedness despite a need for social connection (attachment anxiety; Rohmann et al., 2012) should show negative mental health associations that are stronger in Japan than in Germany. However, as a competing hypothesis, experiential and behavioral patterns that are culturally *incongruent* could have more negative effects on mental health (Curhan et al., 2014). Vulnerable narcissism is incongruent to the prevalent self-construal of Western cultures, where it is more strongly associated with psychological maladjustment than grandiose narcissism. A similar pattern of incongruency and maladjustment might be expected for grandiose narcissism in Eastern cultures. This study hence undertakes a test of competing assumptions regarding the relations between narcissism, self-construal, and mental health.

### 1.6 The present research

We investigate grandiose and vulnerable narcissism in a cross-cultural comparison between Germany and Japan, countries with more independent and more interdependent self-construal (Markus & Kitayama, 1991, 2010). We hypothesize that (1) grandiose narcissism should be higher in Germany than in Japan, whereas vulnerable narcissism should be higher in Japan than in Germany. These differences should be attributable to independent and interdependent self-construal. Given that grandiose and vulnerable narcissism can be described as agentic-antagonistic and neurotic-antagonistic forms of narcissism in FFM terms, and these broad traits differ between cultures (e.g., Schmitt et al., 2007), we want to rule out the possibility that differences in grandiose and vulnerable narcissism might merely reflect broad FFM trait differences. We thus also test whether (2) self-construal would relate to narcissism beyond associations with broad personality traits. Lastly, (3) we test two competing hypotheses on the differential relation of the two forms of narcissism and psychological maladjustment across cultures.

As a precursor to these tests, we investigate the internal structure of grandiose and vulnerable narcissism measures across countries using tests of measurement invariance. While strict assumptions need to be met in order to attribute differences in observed test scores to the same latent dimension, these assumptions are not commonly tested (Fischer & Karl, 2019), and if tested, not always met in cross-cultural personality research. We do thus not expect grandiose and vulnerable narcissism to be fully invariant across cultures. Instead, we consider the level of attained invariance in the interpretation of results, acknowledging that grandiose and vulnerable narcissism might differ in their internal structures and nomological networks across cultures.

## 2 Method

### 2.1 Sample size estimation

To detect mean differences of about one third *SD* (*d* = 0.36; Jonason et al., 2020) and correlation differences > Δ*r* = 0.25 at a power of 1 – β = 0.95, a minimum sample size of *n* = 213 per country or *N* = 426 in total was required.

### 2.2 Participants and procedure

Participants in both countries took part in an online survey (administered via limesurvey; www.limesurvey.org) in either German or Japanese. We recruited students from diverse majors (convenience sampling) via the universities’ mailing lists and offered course credit. Participants were eligible for the study if they were either born in Germany/Japan or lived there at least since the beginning of adolescence (age of thirteen or younger). IRB approval was obtained (EK236052019).

The final sample consisted of *N* = 538 individuals, thereof *n* = 258 German and *n* = 280 Japanese participants. Participants in the German sample were on average 24.61 (*SD* = 5.33) years old; 63.57% were female, 36.43% male. The median study completion time was 28 min. Participants in the Japanese sample were on average 19.10 (*SD* = 0.76) years old; 50.34% were female, 49.64% male. The median completion time was 27 min. Supplement S1 provides detailed sample characteristics. As the samples differed on age and gender, we controlled these variables in all subsequent analyses.^3^

### 2.3 Measures

#### 2.3.1 Self-construal

We assessed independent and interdependent self-construal using the 30-item extended version of the Self-Construal Scale (SCS; Singelis, 1994), to be comparable to previous research (Konrath et al., 2009; Rohmann et al., 2012). Translations and back-translations to English were performed by the authors, supported by a German/Japanese and by an English/Japanese bilingual, both familiar with personality research methods. The internal consistencies for independence and interdependence were α = 0.68/α = 0.68 in the German sample, and α = 0.67/α = 0.79 in the Japanese sample, similar to the original publication (Singelis, 1994).

#### 2.3.2 Grandiose narcissism

We assessed grandiose narcissism using the 13-item version of the Narcissistic Personality Inventory (NPI-13; Gentile et al., 2013). This short version allows assessing the factors leadership/authority, grandiose exhibitionism, and entitlement/exploitativeness, proposed by Ackerman et al. (2011). German and Japanese versions were obtained from Magdalena Żemojtel-Piotrowska/University of Gdansk and previously used in cross-cultural research (Żemojtel-Piotrowska et al., 2019). Different from previous research, NPI-13 items (high-narcissism response option of the original forced choice items) were answered on a 7-point Likert-type scale to facilitate confirmatory factor analyses (Żemojtel-Piotrowska et al., 2019). The internal consistency of the NPI-13 was α = 0.84 for the German and α = 0.79 for the Japanese sample. Internal consistencies for the factors were α = 0.82/α = 0.71, α = 0.74/α = 0.70, and α = 0.60/α = 0.44^4^ for leadership/authority, grandiose exhibitionism, and entitlement/exploitativeness in the German and Japanese samples.

#### 2.3.3 Vulnerable narcissism

We assessed vulnerable narcissism using the extended version of the Hypersensitive Narcissism Scale (HSNS; Hendin & Cheek, 1997), the 23-item Maladaptive Covert Narcissism Scale (MCNS; Cheek et al., 2013). The German^5^ and Japanese versions were translated and back-translated to English by the authors, supported by German/Japanese and English/Japanese bilinguals. The internal consistency was α = 0.89 for the German and α = 0.83 for the Japanese sample, indicating good reliability (which was markedly higher than for the original 10-item version; Cheek et al., 2013).

#### 2.3.4 Five-Factor model traits

To assess broad personality dimensions, we used the 50-item International Personality Item Pool FFM scales (IPIP-FFM; Goldberg, 1992; German version by Ostendorf, 2003; Japanese version by Wakabayashi, 2014). Internal consistencies ranged from 0.77 < *α* < 0.91 for the German sample (*M* = 0.84, *SD* = 0.06) and from 0.76 < *α* < 0.86 for the Japanese sample (*M* = 0.80, *SD* = 0.05).

#### 2.3.5 Psychological maladjustment

To investigate *intrapersonal* ^6^ maladjustment, we assessed global symptom load using the 53-item Brief Symptom Inventory (BSI; Derogatis & Melisaratos, 1983; German version by Franke, 2000). The Japanese version was translated and back-translated to English by the authors, supported by a bilingual. The BSI comprises nine subscales spanning various psychological symptoms such as anxiety, fear, depression, or somatization, and can be aggregated to a composite Global Severity Index (GSI). The internal consistency of the GSI was α = 0.96 in the German sample and α = 0.97 in the Japanese sample.

To investigate *interpersonal maladjustment*, we used the Inventory of Interpersonal Problems (IIP; Horowitz et al., 2000; German version by Horowitz et al., 2016; Japanese version by Suzuki & Fujiyama, 2011). The IIP comprises eight scales which can be arranged on a circumplex spanning the axes agency and communion. For this study, we shortened the 64-item version to a 32-item version (same items in both language versions), based on the highest-loading four items per scale in the Japanese version. The internal consistency of the 32-item IIP was α = 0.89 in the German and α = 0.87 in the Japanese sample.

### 2.4 Analysis plan

Prior to our hypotheses tests, we tested measurement invariance of the NPI and MCNS to assess whether these have the same latent structure across cultures. As results indicated that the general factor of the NPI assesses different constructs across cultures, we analyzed lower-order factor scores (for which invariance can be assumed to some extent; see Results) in subsequent analyses. For reasons of consistency, we also display results for the general factor score. We complemented measurement invariance tests by inspecting country-level nomological networks (FFM traits) for both forms of narcissism.

We tested our hypotheses using hierarchical multiple regression models, always controlling participant age and gender (see Participants and procedure) in the first step. To test the hypothesis that (1) grandiose narcissism would be higher in Germany whereas vulnerable narcissism would be higher in Japan, and these differences would be attributable to independent and interdependent self-construal, we used regression models to predict grandiose and vulnerable narcissism by country, and then entered independent and interdependent self-construal in the next step to see whether these might account for effects of country. To test the assumption that (2) self-construal should relate to narcissism beyond associations with broad personality traits, we next entered FFM dimensions in these models. Finally, to test whether (3) the two forms of narcissism would relate differentially to psychological maladjustment, we predicted intrapersonal and interpersonal maladjustment by grandiose and vulnerable narcissism, and tested for interactions with country. Interaction terms were set up using residual centering (Lance, 1988).

## 3 Results

### 3.1 Measurement invariance and nomological networks

We evaluated invariance of the NPI-13 and the MCNS using Confirmatory Factor Analysis models in Mplus 8. For the NPI-13, we first specified a correlated three-factor – model as in previous research (items loading on factors; Żemojtel-Piotrowska et al., 2019). As this model did not display satisfactory data fit, and coefficients indicated that part of the misspecification might stem from the correlations among the factors, we modeled the three factors separately. Here, metric invariance can be assumed for the entitlement/exploitativeness factor, but not for the other factors (see Supplement S2). The nomological network in terms of correlations with relevant FFM traits were largely as expected and similar across countries for extraversion, but differed from expectations regarding agreeableness, which was negatively related (as expected; Miller et al., 2016) only in Germany, but not in Japan (see Supplement S1). For the MCNS, considering the large number of items, we used four item parcels. Results show that metric invariance can be assumed. The nomological network was mostly similar for the MCNS across Germany and Japan, pointing to an emotionally instable, disagreeable, and introverted personality profile (Jauk et al., 2017; Miller et al., 2016).^8^

### 3.2 Hypothesis 1: grandiose and vulnerable narcissism across cultures

Table 1 displays predictors of grandiose and vulnerable narcissism across samples. We observed a trend (β = – 0.09) for the overall score of grandiose narcissism to be higher in Germany than in Japan (see Table 1, Step 2). A pronounced difference was evident for the grandiose exhibitionism factor (β = – 0.29). Contrary to our expectations, these effects were not dependent upon self-construal: taking up self-construal as predictors did not alter the results, though independent self-construal, as expected, was substantially related to grandiose narcissism and its lower-order-factors (see Step 3). Note, however, that none of the grandiose narcissism scores except entitlement/exploitativeness were invariant, and thus likely reflect different constructs across cultures. Interestingly and unexpectedly, we observed higher entitlement/exploitativeness scores in Japan than in Germany (β = 0.17). Again, this effect was not attributable to differences in self-construal (see Step 3).

Regarding vulnerable narcissism, in line with our expectations, we observed higher scores in Japan than in Germany (β = 0.22, see Step 2). Contrary to our expectations, this relationship was not attributable to self-construal, and self-construal itself was not related to vulnerable narcissism (weakly related in the German sample, and unrelated in the Japanese sample; see Table S1). However, when controlling for differences in FFM traits, associations with self-construal became evident, as described next.

### 3.3 Hypothesis 2: associations between self-construal and narcissism beyond Five-Factor Model traits

To test whether self-construal and narcissism would be associated above and beyond broad traits, we entered FFM traits as additional predictors to the models described above. As Table 1 (Step 4) shows, independent self-construal was associated with grandiose narcissism and its factors (except grandiose exhibitionism) beyond FFM traits. Also, country-level differences held when controlling for FFM traits, with the difference in entitlement/exploitativeness becoming even stronger. We also observed a country-level difference for leadership/authority now, which was not evident in the previous regression steps.

For vulnerable narcissism, we observed a significant relationship with interdependent self-construal as soon as FFM traits were held constant (β = 0.16, see Step 4). While this is in line with our expectations, we also observed an association with independent self-construal (β = 0.12).

### 3.4 Hypothesis 3: differential relations of narcissism and psychological maladjustment across cultures

**Fig. 1:**
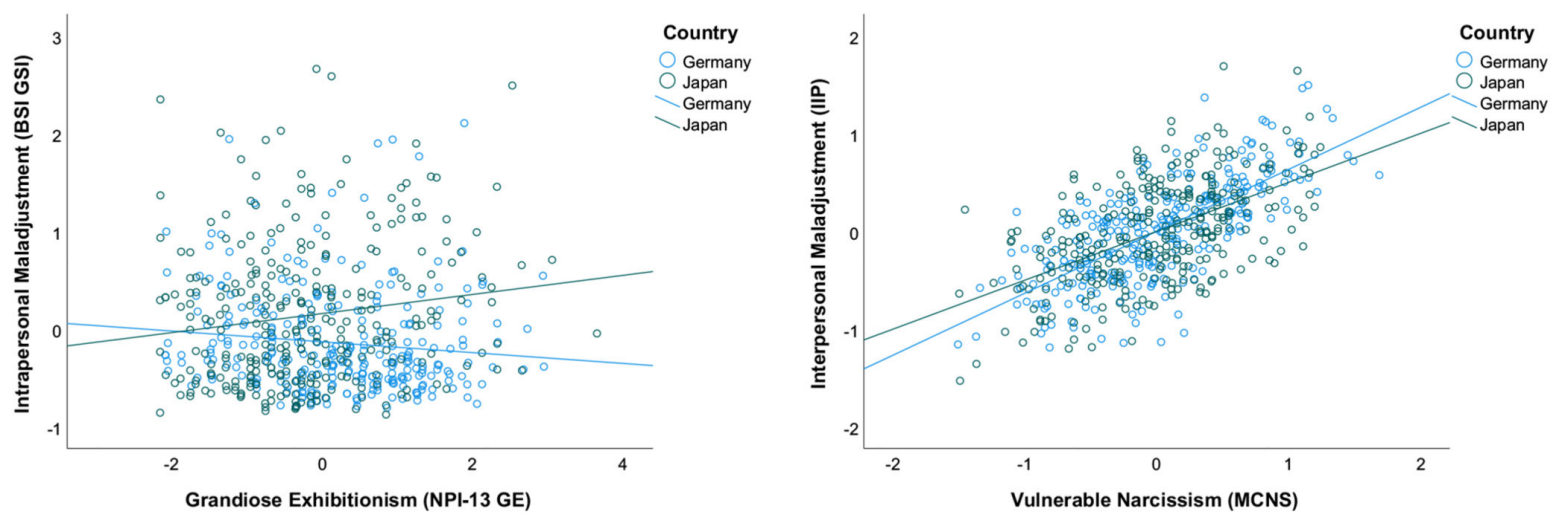
Associations between narcissism measures and intra- (left) as well as interpersonal (right) maladjustment. For reasons of consistency with the correlation and regression results, variables are residualized for age and gender (associations between non-residualized raw scores are only marginally different). NPI = Narcissistic Personality Inventory. MCNS = Maladaptive Covert Narcissism Scale. BSI = Brief Symptom Inventory, GSI = Global Severity Index. IIP = Inventory of Interpersonal Problems.

Table 2 displays the associations of narcissism with intra- and interpersonal maladjustment. Generally, intrapersonal symptoms were higher in Japan than Germany, while there was no difference in interpersonal symptoms (see Step 2). Regarding intrapersonal symptoms, all indicators of grandiose (except grandiose exhibitionism) and vulnerable narcissism displayed significant associations, but the effect of vulnerable narcissism was much stronger. Interpersonal symptoms were strongly related to vulnerable but not grandiose narcissism, with the exception of the entitlement/exploitativeness factor. The crucial test of our hypothesis concerns interactions between narcissism and country (see Step 4). We observed a significant interaction of vulnerable narcissism and country on interpersonal maladjustment, and a significant interaction of grandiose exhibitionism on intrapersonal maladjustment. Fig. 1 displays posttests for these effects, which show that vulnerable narcissism relates more strongly to interpersonal problems in Germany (*r* = 0.72, *p* < .001) than Japan (*r* = 0.54, *p* < .001), whereas grandiose exhibitionism goes along with more intrapersonal symptoms in Japan (*r* = 0.14, *p* < .05), but less in Germany (*r* = – 0.16, *p* < .05).

## 4 Discussion

We investigated grandiose and vulnerable narcissism across Germany and Japan, two countries differing in independent and interdependent self-construal. We tested whether (1) grandiose narcissism would be higher in Germany, whereas vulnerable narcissism would be higher in Japan, and that (2) these differences would relate to selfconstrual beyond broad FFM traits. Finally, (3) we tested two competing hypotheses regarding the relations between narcissism and psychological maladjustment across independent and interdependent cultures.

### 4.1 Vulnerable narcissism has a similar structure, yet different implications across cultures

Results largely confirmed our expectations for vulnerable narcissism, which was (1) higher in Japan than Germany, (2) related to interdependent self-construal beyond FFM traits (albeit also related to independent self-construal) and (3) related more strongly to interpersonal problems in Germany than Japan, which is in line with the cultural incongruency hypothesis on personality and mental health (Curhan et al., 2014). This latter result suggests that, while vulnerable narcissism goes along with interpersonal problems in both cultures, the burden for individuals high on vulnerable narcissism might be higher in a cultural context valuing individualism and assertiveness. The MCNS as a measure of vulnerable narcissism displayed metric invariance, which means that indicators loaded equally on a latent factor (however, intercepts differed). Nomological network structure within the FFM was similar for the central dimensions of neuroticism and disagreeableness (Miller et al., 2016) as well as introversion (Jauk et al., 2017).

### 4.2 Grandiose narcissism has different structures across cultures, but entitlement might be similar

For grandiose narcissism, the measure used in this study (NPI-13) was not invariant at a general factor level (similar to previous research; Żemojtel-Piotrowska et al., 2019), so we conducted analyses for lower-order factors. Here, the entitlement/exploitativeness-factor displayed metric invariance, the others did not. Though this result is at odds with a recent study by Żemojtel-Piotrowska and colleagues, who observed invariance for the other two factors (leadership/authority and grandiose exhibitionism; Żemojtel-Piotrowska et al., 2019), it fits conceptually with structural models of narcissism placing entitlement – an aspect of antagonism – at the core of the construct (Krizan & Herlache, 2018; Weiss et al., 2019).

Contrary to our expectations, the entitlement aspect of narcissism was (1) higher in Japan than Germany (even more when controlling for FFM traits) and (2) controlling for self-construal did not alter this difference. While different post-hoc explanations for this finding could be conceived, when considered together with FFM differences observed here, it most likely reflects a reference group effect (see Limitations). Grandiose exhibitionism, the more (though not exclusively) agentic-extraverted aspect of grandiose narcissism was, in line with our expectations, lower in Japan (note, however, that this aspect likely assesses different constructs between cultures). This latter aspect, which is arguably most culturally incongruent with the Japanese culture, (3) was related to intrapersonal maladjustment in Japan, but not in Germany, further confirming the cultural incongruency hypothesis (Curhan et al., 2014). This shows that, while more agentic narcissism is largely associated with good mental health (less symptoms) in Western samples (e. g., Kaufman et al., 2018), this allegedly “happy face” (Rose, 2002) imposes a burden on the individual in cultures which value modesty and relatedness.

### 4.3 Limitations

An important methodological limitation of this study is that we relied on self-reports within the investigated cultures, in which cross-cultural differences might be obscured by reference group effects (Heine et al., 2002). This was likely the case for (part of) the selfconstrual scale, which showed an expected difference only for interdependent but not independent self-construal (despite experts’ general agreement on independent orientation being very untypical for Japan; ibid.). Also, the scale displays limited reliability for its length. Regarding narcissism, while most of the effects observed here were in line with theoretical predictions, making reference group effects unlikely in these cases, the higher entitlement score in Japan might reflect such an effect, as do differences in FFM traits (see Supplement S1): as in previous research, Japanese participants rated themselves lower on agreeableness and conscientiousness than Germans, which might rather be indicative of high within-culture comparison standards than actual between-culture effects (Schmitt et al., 2007).

Another potential limitation could be seen in non-invariance of the grandiose narcissism measure/imperfect invariance of the vulnerable narcissism measure and entitlement scale. However, we wish to emphasize that we consider the finding that the complex psychological phenomenon of grandiose narcissism – rooted in Western thinking – varies across fundamentally different cultures an important insight rather than a “lack of invariance”. Nonetheless, when interpreting the findings presented here, it must be taken into account that vulnerable narcissism and entitlement do *only partially reflect the same latent constructs* across cultures, and leadership/authority and grandiose exhibitionism *likely reflect different constructs* and must be interpreted at the level of observed test scores (with varying meanings).

## 5 Conclusion

Grandiose and vulnerable narcissism show considerable variation across more independent and interdependent cultures, both in their internal structure and external correlates. Vulnerable narcissism, as expected, was higher in Japan than Germany, but mental health problems related to it were higher in Germany. Grandiose exhibitionism, a more agentic aspect of grandiose narcissism, was higher in Germany and more strongly associated with mental health problems in Japan. These findings support the cultural incongruency-hypothesis on personality and mental health and put the adaptive- or maladaptiveness of narcissism into a cultural perspective.

## Supplementary Material

Supplementary data to this article can be found online at https://doi.org/10.1016/j.paid.2021.110716.

Supplementary Materials

## Figures and Tables

**Table 1:** Associations of country, self-construal, and broad personality traits with narcissism measures.

	Grandiose narcissism				Vulnerable narcissism
	NPI overall	NPI LA	NPI GE	NPI EE	MCNS
*Step 1*					
Age	**−0.10** (−0.18 to −0.01)	− 0.02 (−0.1–0.07)	−0.03 (−0.11–0.06)	**−0.20** (−0.28 to −0.12)	**−0.25** (−0.33 to −0.17)
Gender	**0.10** (0.02–0.19)	**0.14** (0.05–0.22)	0.03 (−0.06–0.11)	**0.09** (0.01–0.17)	0.00 (−0.09–0.08)
	*R* ^2^_adj_ = **0.02**	*R* ^2^_adj_ = **0.02**	*R* ^2^_adj_ = 0.00	*R* ^2^_adj_ = **0.05**	*R* ^2^_adj_ = **0.06**
*Step 2*					
Age	**−0.15** (−0.26 to −0.05)	−0.04 (−0.15–0.06)	**−0.20** (−0.30 to −0.10)	*−0.10* (−0.20–0.00)	**−0.12** (−0.22 to −0.02)
Gender	**0.12** (0.03–0.20)	**0.14** (0.06–0.23)	0.07 (−0.02-0.15)	0.07 (−0.02-0.15)	−0.03 (−0.12–0.05)
Country	*−0.09* (−0.20–0.01)	−0.04 (−0.15–0.06)	**−0.29** (−0.40 to −0.19)	**0.17** (0.07–0.28)	**0.22** (0.12–0.32)
	*R* ^2^_adj_ = **0.02**	*R* ^2^_adj_ = **0.02**	*R* ^2^_adj_ = **0.05**	*R* ^2^_adj_ = **0.06**	*R* ^2^_adj_ = **0.09**
*Step 3*					
Age	**−0.17** (−0.27 to −0.07)	−0.07 (−0.17–0.03)	**−0.21** (−0.31 to −0.11)	**−0.12** (−0.22 to −0.02)	**−0.11** (−0.21 to −0.01)
Gender	**0.09** (0.01–0.17)	**0.11** (0.03–0.19)	0.05 (−0.03–0.13)	0.04 (−0.04–0.12)	−0.03 (−0.11–0.05)
Country	*−0.10* (−0.20–0.00)	−0.04 (−0.14–0.06)	**−0.30** (−0.41 to −0.20)	**0.17** (0.07–0.27)	**0.22** (0.11–0.32)
Self-construal					
Independent	**0.35** (0.27–0.43)	**0.33** (0.25–0.41)	**0.25** (0.17–0.33)	**0.25** (0.17–0.33)	−0.04 (−0.13–0.04)
Interdependent	−0.03 (−0.11–0.05)	*-0.07* (−0.15–0.01)	0.02 (−0.06–0.11)	−0.04 (−0.12–0.05)	0.02 (−0.06–0.11)
	*R* ^2^_adj_ = **0.14**	*R* ^2^_adj_ = **0.13**	*R* ^2^_adj_ = **0.11**	*R* ^2^_adj_ = **0.12**	*R* ^2^_adj_ = **0.09**
*Step 4*					
Age	**−0.15** (−0.24 to −0.06)	−0.05 (−0.14–0.04)	**−0.20** (−0.29 to −0.10)	**−0.10** (−0.19 to −0.01)	**−0.10** (−0.17 to −0.02)
Gender	*0.07* (0.00–0.15)	**0.09** (0.01–0.16)	0.05 (−0.03–0.14)	0.03 (−0.05–0.11)	−0.05 (−0.12–0.02)
Country	0.09 (−0.02–0.20)	**0.16** (0.05–0.27)	**−0.16** (−0.27 to −0.04)	**0.25** (0.14–0.37)	**0.10** (0.01–0.20)
Self-construal					
Independent	**0.14** (0.06–0.22)	**0.10** (0.01–0.18)	0.07 (−0.02–0.16)	**0.17** (0.09–0.26)	**0.12** (0.05–0.20)
Interdependent	0.02 (−0.07–0.10)	−0.01 (−0.10–0.07)	0.01 (−0.08–0.10)	0.04 (−0.05–0.13)	**0.16** (0.09–0.23)
FFM traits					
Emotional Stability	**−0.14** (−0.21 to –0.06)	*−0.07* (−0.15–0.01)	−0.01 (−0.09–0.07)	**−0.27** (−0.35 to −0.19)	**-0.55** (−0.62 to −0.49)
Extraversion	**0.36** (0.27–0.44)	**0.38** (0.29–0.46)	**0.29** (0.20–0.38)	**0.17** (0.08–0.25)	**−0.16** (−0.23 to −0.08)
Openness	**0.34** (0.25–0.44)	**0.36** (0.26–0.45)	**0.20** (0.09–0.30)	**0.27** (0.17–0.37)	**0.20** (0.12–0.29)
Agreeableness	**−0.13** (−0.23 to −0.04)	**−0.16** (−0.25 to −0.07)	0.01 (−0.09–0.11)	**−0.19** (−0.29 to −0.09)	**−0.24** (−0.32 to −0.16)
Conscientiousness	0.02 (−0.05–0.10)	0.02 (−0.05–0.10)	−0.04 (−0.13–0.04)	**0.08** (0.01–0.16)	**−0.07** (−0.14–0.00)
	*R* ^2^_adj_ = **0.32**	*R* ^2^_adj_ = **0.30**	*R* ^2^_adj_ = **0.20**	*R* ^2^_adj_ = **0.25**	*R* ^2^_adj_ = **0.48**

Note. Coefficients in bold type are significant at *p* < .05, coefficients in italic type reflect trends at *p* < .10; parentheses denote 95% *CI*. NPI = Narcissistic Personality Inventory, LA = leadership/authority, GE = grandiose exhibitionism, EE = entitlement/exploitativeness. MCNS = Maladaptive Covert Narcissism Scale. FFM = Five-Factor Model. Gender was coded 0 = female and 1 = male. Country was coded 0 = Germany and 1 = Japan.

**Table 2:** Country-specific associations between narcissism measures and psychological maladjustment.

	Intrapersonal maladjustment (BSI GSI)					Interpersonal maladjustment (IIP)				
*Step 1*										
Age	**−0.24**					−0.05				
	(−0.33 to −0.16)					(−0.14–0.03)				
Gender	0.05					0.03				
	(−0.03–0.13)					(−0.05–0.12)				
	*R* ^2^_adj_ = **0.06**					*R* ^2^_adj_ = 0.00				
*Step 2*										
Age	−0.05					−0.04				
	(−0.15–0.05)					(−0.14–0.07)				
Gender	0.01					0.03				
	(−0.07–0.09)					(−0.06–0.11)				
Country	**0.33**					0.03				
	(0.23–0.43)					(−0.08–0.13)				
	*R* ^2^_adj_ = **0.12**					*R* ^2^_adj_ = 0.00				
*Step 3*	NPI overall	NPI LA	NPI GE	NPI EE	MCNS	NPI overall	NPI LA	NPI GE	NPI EE	MCNS
Age	−0.03	−0.05	−0.04	−0.04	0.01	−0.04	−0.04	−0.04	−0.03	0.04
	(−0.13–0.07)	(−0.15–0.05)	(−0.14–0.06)	(−0.13–0.06)	(−0.07–0.10)	(−0.14–0.07)	(−0.14–0.07)	(−0.15–0.06)	(−0.13–0.08)	(−0.04–0.12)
Gender	0.00	0.00	0.00	0.00	0.02	0.03	0.03	0.03	0.02	0.05
	(−0.08–0.08)	(−0.08–0.08)	(−0.08–0.09)	(−0.08–0.08)	(−0.04–0.09)	(−0.06–0.11)	(−0.05–0.12)	(−0.06–0.12)	(−0.06–0.11)	(−0.02–0.12)
Country	**0.34**	**0.33**	**0.34**	**0.30**	**0.22**	0.03	0.02	0.01	0.01	**−0.12**
	(0.24–0.44)	(0.23–0.43)	(0.24–0.44)	(0.20–0.40)	(0.13–0.30)	(−0.08–0.13)	(−0.08–0.13)	(−0.10–0.12)	(−0.10–0.12)	(−0.20 to −0.03)
Narcissism measure	**0.10**	*0.07*	0.05	**0.14**	**0.51**	0.00	−0.05	−0.04	**0.10**	**0.66**
	(0.02–0.18)	(−0.01–0.15)	(−0.03–0.13)	(0.06–0.22)	(0.44–0.58)	(−0.09–0.08)	(−0.13–0.04)	(−0.13–0.05)	(0.01–0.19)	(0.59–0.73)
	*R* ^2^_adj_ = **0.13**	*R* ^2^_adj_ = **0.13**	*R* ^2^_adj_ = **0.12**	*R* ^2^_adj_ = **0.14**	*R* ^2^_adj_ = **0.36**	*R* ^2^_adj_ = 0.00	*R* ^2^_adj_ = 0.00	*R* ^2^_adj_ = 0.00	*R* ^2^_adj_ = 0.01	*R* ^2^_adj_ = **0.39**
*Step 4*										
Age	−0.05	−0.05	−0.06	−0.04	0.01	−0.04	−0.04	−0.06	−0.02	0.05
	(−0.15–0.05)	(−0.15–0.05)	(−0.17–0.04)	(−0.13–0.06)	(−0.08–0.09)	(−0.15–0.07)	(−0.15–0.07)	(−0.17–0.05)	(−0.13–0.08)	(−0.03–0.13)
Gender	0.00	0.00	0.01	0.00	0.02	0.03	0.03	0.03	0.02	0.05
	(−0.08–0.08)	(−0.09–0.08)	(−0.07–0.09)	(−0.08–0.08)	(−0.05–0.09)	(−0.06–0.11)	(−0.05–0.12)	(−0.05–0.12)	(−0.07–0.11)	(−0.01–0.12)
Country	**0.33**	**0.33**	**0.32**	**0.30**	**0.21**	0.02	0.02	0.00	0.01	**−0.11**
	(0.23–0.43)	(0.23–0.43)	(0.22–0.43)	(0.20–0.40)	(0.13–0.30)	(−0.08–0.13)	(−0.08–0.13)	(−0.11–0.11)	(−0.10–0.12)	(−0.20 to −0.03)
Narcissism measure	**0.10**	*0.07*	0.04	**0.14**	**0.51**	0.00	−0.05	−0.04	**0.10**	**0.66**
	(0.02–0.18)	(−0.01–0.15)	(−0.04–0.13)	(0.06–0.22)	(0.44–0.58)	(−0.09–0.08)	(−0.13–0.04)	(−0.13–0.05)	(0.01–0.19)	(0.59–0.73)
Narcissism measure * country	*0.07*	0.06	**0.11**	0.00	0.02	0.02	0.02	*0.08*	−0.05	**−0.08**
	(0.00–0.15)	(−0.02–0.14)	(0.03–0.19)	(−0.08–0.08)	(−0.04–0.09)	(−0.06–0.11)	(−0.07–0.10)	(−0.01–0.16)	(−0.13–0.03)	(−0.15 to −0.02)
	*R* ^2^_adj_ = **0.14**	*R* ^2^_adj_ = **0.13**	*R* ^2^_adj_ = **0.13**	*R* ^2^_adj_ = **0.14**	*R* ^2^_adj_ = **0.36**	*R* ^2^_adj_ = 0.00	*R* ^2^_adj_ = 0.00	*R* ^2^_adj_ = 0.00	*R* ^2^_adj_ = 0.01	*R* ^2^_adj_ = **0.40**

Note. Coefficients in bold type are significant at *p* < .05, coefficients in italic type reflect trends at *p* < .10; parentheses denote 95% *CI*. BSI = Brief Symptom Inventory, GSI = Global Severity Index. IIP = Inventory of Interpersonal Problems. NPI = Narcissistic Personality Inventory, LA = leadership/authority, GE = grandiose exhibitionism, EE = entitlement/exploitativeness. MCNS = Maladaptive Covert Narcissism Scale. Gender was coded 0 = female and 1 = male. Country was coded 0 = Germany and 1 = Japan.

## Footnotes

1 While there is strong agreement by cultural experts on such differences (Heine et al., 2002), those do not always reflect in self-ratings of individuals from the respective cultures due to *reference group effects* (comparison standards for self-ratings within cultural groups; ibid.). Thus, we use ratings of selfconstrual as an explanatory variable in this study, but place our main emphasis on country-level differences.

3 Analyses without controlling for age and gender yielded the same pattern of results; see Supplements S3 and S4.

4 We note that this four-item-scale displays low internal consistency in the Japanese sample. However, a latent model (see Results and Supplement S2) turned out to be satisfactory.

5 The 10 German HSNS items were taken from Jauk et al. (2017).

6 Note that the BSI assesses primarily intrapersonal symptoms, but also comprises symptoms related to social situations. For reasons of comparability to previous research, we retained the respective scales in the BSI. Complemental analyses showed that the results reported here (Table 2) are not affected by whether these scales are included or not.

8 Note, however, that both grandiose and vulnerable narcissism differed in correlations with other FFM traits which are not commonly considered as relevant in structural models of narcissism (Miller et al., 2016; Weiss et al., 2019). Supplement S1 displays the full correlation matrix.
