# Supplementary material for: Narcissism in independent and interdependent cultures
Source: Pers Individ Dif. Author manuscript; Available in PMC 2021 Jul 19. (PMC7611310; doi:10.1016/j.paid.2021.110716)
Supplement: Supplementary Materials [file EMS130372-supplement-Supplementary_Materials.zip › 1-s2.0-S019188692100091X-mmc1.docx]

Table S1. Descriptive statistics and intercorrelations of the study variables

|  | *M* (*SD*) | 3 | 4 | 5 | 6 | 7 | 8 | 9 | 10 | 11 | 12 | 13 | 14 | 15 | 16 |
| --- | --- | --- | --- | --- | --- | --- | --- | --- | --- | --- | --- | --- | --- | --- | --- |
| *Demographics* | | | | | | | | | | | | | | | |
| Age (1) | 24.62 (5.33)  19.10 (0.76) |  |  |  |  |  |  |  |  |  |  |  |  |  |  |
| Gender (2) | 0.36 (0.48)  0.50 (0.50) |  |  |  |  |  |  |  |  |  |  |  |  |  |  |
|  | | | | | | | | | | | | | | | |
| *Grandiose Narc. Overall (NPI, 3)* | 3.24 (0.91)  3.26 (0.90) |  | **.83** | **.76** | **.83** | **.14** | **.44** | -.06 | .07 | **.45** | **.32** | -.04 | -.01 | .04 | -.01 |
| NPI LA (4) | 3.12 (1.21)  3.12 (1.25) | **.84** |  | **.36** | **.66** | .06 | **.39** | -.07 | **.16** | **.41** | **.34** | -.05 | .05 | .01 | -.05 |
| NPI GE (5) | 3.28 (1.09)  2.91 (1.11) | **.78** | **.45** |  | **.40** | -.03 | **.36** | .03 | **.13** | **.41** | **.19** | .08 | -.06 | -.08 | *-.11* |
| NPI EE (6) | 3.30 (1.09)  3.83 (1.06) | **.76** | **.57** | **.33** |  | **.35** | **.32** | *-.12* | **-.14** | **.23** | **.26** | **-.16** | .00 | **.19** | **.16** |
|  | | | | | | | | | | | | | | | |
| *Vulnerable Narc. (MCNS, 7)* | 2.69 (0.59)  3.04 (0.57) | **.35** | **.25** | **.18** | **.43** |  | **-.19** | *.11* | **-.62** | **-.32** | *-.10* | **-.26** | **-.23** | **.63** | **.72** |
| *Self-Construal* | | | | | | | | | | | | | | | |
| Independence (8) | 4.51 (0.62)  4.52 (0.67) | **.28** | **.29** | **.18** | **.21** | .07 |  | -.07 | **.24** | **.57** | **.37** | **.13** | **.13** | **-.14** | **-.33** |
| Interdependence (9) | 4.50 (0.59)  4.76 (0.77) | -.04 | -.08 | -.01 | .01 | -.02 | -.01 |  | .01 | -.03 | **-.18** | **.40** | .01 | .06 | **.17** |
| *FFM Traits* | | | | | | | | | | | | | | | |
| Emotional Stability (10) | 2.97 (0.74)  2.63 (0.82) | **-.13** | -.08 | -.01 | **-.26** | **-.56** | **.13** | -.07 |  | **.22** | *.12* | .09 | **.26** | **-.55** | **-.50** |
| Extraversion (11) | 2.99 (0.78)  2.84 (0.85) | **.43** | **.46** | **.35** | **.19** | **-.15** | **.40** | **.12** | **.15** |  | **.26** | **.26** | .01 | **-.25** | **-.41** |
| Openness (12) | 3.93 (0.47)  3.04 (0.68) | **.43** | **.42** | **.30** | **.31** | **.23** | **.27** | -.06 | -.05 | **.25** |  | .08 | .09 | *-.11* | **-.25** |
| Agreeableness (13) | 4.00 (0.55)  3.64 (0.64) | **.14** | .09 | **.18** | .05 | **-.13** | **.16** | **.50** | -.03 | **.35** | **.22** |  | *.12* | *-.11* | -.16 |
| Conscientiousness (14) | 3.63 (0.57)  3.27 (0.71) | *.11* | .07 | .06 | **.14** | -.09 | **.15** | **.20** | .08 | *.12* | *.10* | **.19** |  | **-.23** | **-.29** |
| *Symptoms* | | | | | | | | | | | | | | | |
| Intrapersonal (BSI GSI, 15) | 0.51 (0.49)  0.98 (0.72) | **.15** | *.11* | **.14** | **.12** | **.46** | -.07 | .01 | **-.37** | -.08 | **.17** | -.09 | *-.11* |  | **.60** |
| Interpersonal (IIP, 16) | 1.39 (0.53)  1.45 (0.53) | .01 | -.05 | .03 | .04 | **.54** | **-.20** | .01 | **-.33** | **-.36** | .04 | **-.24** | **-.24** | **.54** |  |

*Note*. Coefficients for the German sample are displayed in the upper row / above the diagonal, coefficients for the Japanese sample in the lower row / below the diagonal. NPI = Narcissistic Personality Inventory, LA = leadership/authority, GE = grandiose exhibitionism, EE = entitlement/exploitativeness. MCNS = Maladaptive Covert Narcissism Scale. BSI = Brief Symptom Inventory, GSI = Global Severity Index. IIP = Inventory of Interpersonal Problems. All means differ significantly at *p* < .05, except NPI Overall (*p* = .77), NPI LA (*p* = .96), Independence (*p* = .84), and Interpersonal Problems (*p* = .24). Correlation coefficients reflect partial correlations controlling for age and gender. Gender was coded 0 = female and 1 = male. Coefficients in bold type are significant at *p* < .05, coefficients in italic type reflect trends at *p* < .10.

The correlation patterns among the NPI total score and its factors were generally similar in the German and Japanese samples. The correlation between grandiose and vulnerable narcissism was within the expected small positive range (e.g., Jauk et al., 2017) in the German sample, but was higher in the Japanese sample. At a factor level, only entitlement displayed a positive correlation with vulnerable narcissism in the German sample, whereas all factors displayed positive correlations in the Japanese sample. As expected, grandiose narcissism was associated with independent self-construal in both samples, but different from expectations, there was no association between vulnerable narcissism and interdependent self-construal. Associations between grandiose narcissism and FFM traits were generally similar in both countries, with strongest positive associations for extraversion and openness, but a theoretically expected negative correlation (e.g., Miller et al., 2016; Weiss et al., 2019) between grandiose narcissism and agreeableness was present only for the EE factor in the German sample, not in the Japanese sample. The FFM dimensions themselves generally displayed low-to-moderate intercorrelations in both samples. Finally, the measures of intra- and interpersonal symptoms displayed substantial and similar intercorrelation in both samples, and were substantially negatively related to emotional stability, and positively related to vulnerable narcissism in both samples. Section 3.4 explores these relations in greater detail.
