# Supplementary material for: Narcissism in independent and interdependent cultures
Source: Pers Individ Dif. Author manuscript; Available in PMC 2021 Jul 19. (PMC7611310; doi:10.1016/j.paid.2021.110716)
Supplement: Supplementary Materials [file EMS130372-supplement-Supplementary_Materials.zip › 1-s2.0-S019188692100091X-mmc2.docx]

Table S2. Invariance tests for the narcissism measures

|  | Model fit | Δ χ^2^ |
| --- | --- | --- |
|  |  |  |
| *Grandiose narcissism* |  |  |
| Leadership/authoritiy |  |  |
| Configural | χ^2^_(4)_ = 36.46, *p* < .001; CFI = 0.95; RMSEA= 0.17 (*p*_RMSEA<.05_ = .00); SRMR= 0.04) | - |
| Metric | χ^2^_(7)_ = 46.04, *p* < .001; CFI = 0.94; RMSEA= 0.14 (*p*_RMSEA<.05_ = .00); SRMR= 0.06) | χ^2^_(3)_ = 9.57, *p* = .02 |
| Scalar | χ^2^_(10)_ = 334.90, *p* < .001; CFI = 0.49; RMSEA= 0.35 (*p*_RMSEA<.05_ = .00); SRMR= 0.18) | χ^2^_(3)_ = 288.86, *p* = .00 |
| Grandiose exhibitionism |  |  |
| Configural | χ^2^_(10)_ = 39.60, *p* < .001; CFI = 0.96; RMSEA= 0.11 (*p*_RMSEA<.05_ = .00); SRMR= 0.04) | - |
| Metric | χ^2^_(14)_ = 49.55, *p* < .001; CFI = 0.95; RMSEA= 0.10 (*p*_RMSEA<.05_ = .00); SRMR= 0.06) | χ^2^_(4)_ = 9.98, *p* = .04 |
| Scalar | χ^2^_(18)_ = 189.33, *p* < .001; CFI = 0.75; RMSEA= 0.19 (*p*_RMSEA<.05_ = .00); SRMR= 0.16) | χ^2^_(4)_ = 133.11, *p* = .00 |
| Entitlement/exploitativeness |  |  |
| Configural | χ^2^_(4)_ = 4.23, *p* = .37; CFI = 0.99; RMSEA= 0.02 (*p*_RMSEA<.05_ = .67); SRMR= 0.02) | - |
| Metric | χ^2^_(7)_ = 14.78, *p* = .04; CFI = 0.94; RMSEA= 0.06 (*p*_RMSEA<.05_ = .26); SRMR= 0.05) | χ^2^_(3)_ = 9.56, *p* = .03 |
| Scalar | χ^2^_(10)_ = 55.43, *p* < .001; CFI = 0.66; RMSEA= 0.13 (*p*_RMSEA<.05_ = .00); SRMR= 0.08) | χ^2^_(3)_ = 44.48, *p* = .00 |
|  |  |  |
| *Vulnerable Narcissism* |  |  |
| Configural | χ^2^_(4)_ = 7.93, *p* = .09; CFI = 0.99; RMSEA= 0.06 (*p*_RMSEA<.05_ = .32); SRMR= 0.01) | - |
| Metric | χ^2^_(4)_ = 12.98, *p* = .07; CFI = 0.96; RMSEA= 0.06 (*p*_RMSEA<.05_ = .36); SRMR= 0.05) | χ^2^_(3)_ = 5.05, *p* = .17 |
| Scalar | χ^2^_(10)_ = 103.27, *p* = .00; CFI = 0.92; RMSEA= 0.19 (*p*_RMSEA<.05_ = .00); SRMR= 0.12) | χ^2^_(3)_ = 90.29, *p* = .00 |

*Note*. We evaluated whether the configural invariance model displayed acceptable data fit, and whether imposing additional constraints for metric and scalar models significantly worsened fit. Complemental to that, we also evaluated whether the metric models displayed acceptable fit per se, which was the case for entitlement/exploitativeness, and for vulnerable narcissism. None of the scalar models fit the data.
