# Supplementary material for: Narcissism in independent and interdependent cultures
Source: Pers Individ Dif. Author manuscript; Available in PMC 2021 Jul 19. (PMC7611310; doi:10.1016/j.paid.2021.110716)
Supplement: Supplementary Materials [file EMS130372-supplement-Supplementary_Materials.zip › 1-s2.0-S019188692100091X-mmc3.docx]

Table S3. Associations of country, self-construal, and broad personality traits with narcissism measures, not controlling for age and gender.

|  | Grandiose Narcissism | | | | | | | | Vulnerable Narcissism | |
| --- | --- | --- | --- | --- | --- | --- | --- | --- | --- | --- |
|  | NPI Overall | | NPI LA | | NPI GE | | NPI EE | | MCNS | |
| *Step 1* |  | |  | |  | |  | |  | |
| Country | .01 (-.07 – .10) | | 00. (-.08 – .09) | | **-.17** (-.25 – -.08) | | **.24** (.16 – .32) | | **.28** (.20 – .36) | |
|  | *R*^2^_adj_ = .00 | | *R*^2^_adj_ = .00 | | *R*^2^_adj_ = **.03** | | *R*^2^_adj_ = **.06** | | *R*^2^_adj_ = **.08** | |
| *Step 2* |  | |  | |  | |  | |  | |
| Country | .01 (-.07 – .09) | | .01 (-.07 – .09) | | **-.18** (-.26 – -.09) | | **.24** (.16 – .32) | | **.28** (.19 – .36) | |
| Self-Construal |  | |  | |  | |  | |  | |
| Independent | **.35** (.27 – .43) | | **.33** (.25 – .41) | | **.25** (.17 – .33) | | **.25** (.17 – .33) | | -.05 (-.13 – .03) | |
| Interdependent |  | -.02 (-.10 – .06) |  | *-.07* (-.16 – .01) |  | .04 (-.04 – .12) |  | -.03 (-.11 – .05) |  | .04 (-.05 – .12) |
|  | *R*^2^_adj_ = **.12** | | *R*^2^_adj_ = **.12** | | *R*^2^_adj_ = **.09** | | *R*^2^_adj_ = **.12** | | *R*^2^_adj_ = **.08** | |
| *Step 3* |  | |  | |  | |  | |  | |
| Country | **.18** (.09 – .28) | | **.20** (.10 – .30) | | -.04 (-.14 – .07) | | **.31** (.21 – .42) | | **.15** (.06 – .23) | |
| Self-Construal |  | |  | |  | |  | |  | |
| Independent | **.14** (.06 – .22) | | **.10** (.02 – .19) | | .07 (-.02 – .16) | | **.17** (.09 – .26) | | **.11** (.04 – .19) | |
| Interdependent | .04 (-.05 – .12) | | .00 (-.12 – .12) | | .04 (-.05 – .13) | | .05 (-.04 – .14) | | **.16** (.09 – .23) | |
| FFM Traits |  | |  | |  | |  | |  | |
| Emotional Stability | **-.13** (-.21 – -.06) | | -.06 (-.13 – .02) | | -.01 (-.09 – .07) | | **-.27** (-.35 – -.20) | | **-.56** (-.63 – -.50) | |
| Extraversion | **.36** (.28 – .45) | | **.37** (.29 – .46) | | **.30** (.21 – .39) | | **.17** (.08 – .26) | | **-.15** (-.22 – -.07) | |
| Openness | **.35** (.26 – .45) | | **.37** (.28 – .47) | | **.20** (.10 – .31) | | **.27** (.17 – .37) | | **.19** (.10 – .27) | |
| Agreeableness | **-.16** (-.25 – -.07) | | **-.19** (-.28 – -.10) | | .00 (-.10 – .09) | | **-.20** (-.29 – -.11) | | **-.23** (-.30 – -.15) | |
| Conscientiousness | .01 (-.06 – .09) | | .02 (-.06 – .09) | | -.05 (-.13 – .03) | | **.08** (.00 – .16) | | *-.06* (-.13 – .00) | |
|  | *R*^2^_adj_ = **.30** | | *R*^2^_adj_ = **.30** | | *R*^2^_adj_ = **.18** | | *R*^2^_adj_ = **.25** | | *R*^2^_adj_ = **.47** | |

*Note*. Coefficients in bold type are significant at *p* < .05, coefficients in italic type reflect trends at *p* < .10; parentheses denote 95% *CI*. NPI = Narcissistic Personality Inventory, LA = leadership/authority, GE = grandiose exhibitionism, EE = entitlement/exploitativeness. MCNS = Maladaptive Covert Narcissism Scale. FFM = Five-Factor Model. Country was coded 0 = Germany and 1 = Japan.
