# Supplementary material for: Narcissism in independent and interdependent cultures
Source: Pers Individ Dif. Author manuscript; Available in PMC 2021 Jul 19. (PMC7611310; doi:10.1016/j.paid.2021.110716)
Supplement: Supplementary Materials [file EMS130372-supplement-Supplementary_Materials.zip › 1-s2.0-S019188692100091X-mmc4.docx]

Table S4. Country-specific associations between narcissism measures and psychological maladjustment, not controlling for age and gender.

|  | Intrapersonal Maladjustment (BSI GSI) | | | | | Interpersonal Maladjustment (IIP) | | | | |
| --- | --- | --- | --- | --- | --- | --- | --- | --- | --- | --- |
|  |  |  |  |  |  |  |  |  |  |  |
| *Step 1* |  |  |  |  |  |  |  |  |  |  |
| Country | **.36** (.28 – .44) |  |  |  |  | .05 (-.03 – .14) |  |  |  |  |
|  | *R*^2^_adj_ = **.13** |  |  |  |  | *R*^2^_adj_ = .00 |  |  |  |  |
|  |  |  |  |  |  |  |  |  |  |  |
| *Step 2* | NPI Overall | NPI LA | NPI GE | NPI EE | MCNS | NPI Overall | NPI LA | NPI GE | NPI EE | MCNS |
| Country | **.36** (.28 – .43) | **.36** (.28 – .44) | **.37** (.29 – .45) | **.32** (.24 – .40) | **.21** (.14 – .28) | .05 (-.03 – .14) | .05 (-.03 – .14) | .05 (-.04 – .13) | .03 (-.06 – .11) | **-.13** (-.20 – -.06) |
| Narcissism measure | **.10** (.02 – .18) | *.07* (-.01 – .15) | .05 (-.03 – .13) | **.14** (.06 – .22) | **.51** (.44 – .58) | .00 (-.08 – .09) | -.04 (-.13 – .04) | -.03 (-.12 – .05) | **.10** (.02 – .19) | **.65** (.58 – .72) |
|  | *R*^2^_adj_ = **.14** | *R*^2^_adj_ = **.13** | *R*^2^_adj_ = **.13** | *R*^2^_adj_ = **.14** | *R*^2^_adj_ = **.36** | *R*^2^_adj_ = .00 | *R*^2^_adj_ = .00 | *R*^2^_adj_ = .00 | ***R*^2^_adj_ = .01** | *R*^2^_adj_ = **.39** |
|  |  |  |  |  |  |  |  |  |  |  |
| *Step 3* |  |  |  |  |  |  |  |  |  |  |
| Country | **.36** (.28 – .43) | **.36** (.28 – .44) | **.37** (.29 – .45) | **.32** (.24 – .40) | **.21** (.14 – .28) | .05 (-.03 – .14) | .05 (-.03 – .14) | .05 (-.04 – .13) | .03 (-.06 – .11) | **-.13** (-.20 – -.06) |
| Narcissism measure | **.10** (.02 – .18) | *.07* (-.01 – .15) | .05 (-.03 – .13) | **.14** (.06 – .22) | **.51** (.44 – .58) | .00 (-.08 – .09) | -.04 (-.13 – .04) | -.03 (-.12 – .05) | **.10** (.02 – .19) | **.65** (.58 – .72) |
| Narcissism measure * Country | *.07* (-.01 – .15) | .06 (-.02 – .14) | **.10** (.02 – .18) | .00 (-.08 – .08) | .03 (-.04 – .09) | .02 (-.07 – .10) | .02 (-.07 – .10) | .07 (-.02 – .15) | -.05 (-.14 – .03) | **-.08** (-.14 – -.01) |
|  | *R*^2^_adj_ = **.14** | *R*^2^_adj_ = **.13** | *R*^2^_adj_ = **.14** | *R*^2^_adj_ = **.14** | *R*^2^_adj_ = **.36** | *R*^2^_adj_ = .00 | *R*^2^_adj_ = .00 | *R*^2^_adj_ = .00 | ***R*^2^_adj_ = .01** | *R*^2^_adj_ = **.40** |

*Note*. Coefficients in bold type are significant at *p* < .05, coefficients in italic type reflect trends at *p* < .10; parentheses denote 95% *CI*. BSI = Brief Symptom Inventory, GSI = Global Severity Index. IIP = Inventory of Interpersonal Problems. NPI = Narcissistic Personality Inventory, LA = leadership/authority, GE = grandiose exhibitionism, EE = entitlement/exploitativeness. MCNS = Maladaptive Covert Narcissism Scale. Gender was coded 0 = female and 1 = male. Country was coded 0 = Germany and 1 = Japan.
